# Association between left atrial phasic conduit function and early atrial fibrillation recurrence in patients undergoing electrical cardioversion

**DOI:** 10.1007/s00392-017-1188-9

**Published:** 2017-11-27

**Authors:** Anna Degiovanni, Enrico Boggio, Eleonora Prenna, Chiara Sartori, Federica De Vecchi, Paolo N. Marino, From the Novara Atrial Fibrillation (NAIF) Study Group, Anna Degiovanni, Paolo N. Marino

**Affiliations:** Department of Translational Medicine, Clinical Cardiology, Università del Piemonte Orientale, Azienda Ospedaliera Universitaria “Maggiore della Carità”, Corso Mazzini 18, 28100 Novara, Italy

**Keywords:** Left atrial conduit function, Full-volume 3D echocardiography, Atrial fibrillation, Electrical cardioversion

## Abstract

**Background:**

Diastolic dysfunction promotes atrial fibrillation (AF) inducing left atrial (LA) remodeling, with chamber dilation and fibrosis. Predominance of LA phasic conduit (LAC) function should reflect not only chamber alterations but also underlying left ventricular (LV) filling impairment. Thus, LAC was tested as possible predictor of early AF relapse after electrical cardioversion (EC).

**Methods:**

96 consecutive patients, who underwent EC for persistent non-valvular AF, were prospectively enrolled. Immediately after successful EC (3 h ± 15 min), an echocardiographic apical four-chamber view was acquired with transmitral velocities, annular tissue Doppler and simultaneous LV and LA three-dimensional full-volume datasets. Then, from LA–LV volumetric curves we computed LAC as: [(LV maximum − LV minimum) − (LA maximum − LA minimum) volume], expressed as % LV stroke volume. LA pump, immediately post-EC, was assumed and verified as being negligible. Sinus rhythm persistence at 1 month was checked with ECG-Holter monitoring.

**Results:**

At 1 month 62 patients were in sinus rhythm and 34 in AF. AF patients presented pre-EC higher *E*/*é* values (*p* = 0.012), no major LA volume differences (*p* = NS), but a stiffer LV cavity (*p* = 0.012) for a comparable LV capacitance (*p* = 0.461). Conduit contributed more (*p* < 0.001) to LV stroke volume in AF subpopulation. Multiple regression revealed LAC as the most significant AF predictor (*p* = 0.013), even after correction for biometric characteristics and pharmacotherapy (*p* = 0.008).

**Conclusion:**

Our data suggest that LAC larger contribution to LV filling soon after EC reflects LA–LV stiffening, which skews atrioventricular interaction leading to AF perpetuation and makes conduit dominance a powerful predictor of early AF recurrence.

**Electronic supplementary material:**

The online version of this article (10.1007/s00392-017-1188-9) contains supplementary material, which is available to authorized users.

## Introduction

Atrial fibrillation (AF) is one of the most frequent arrhythmias and one of the major causes of stroke, heart failure and cardiovascular morbidity worldwide [1].

It develops in 1 out of 4 middle-age adults in Europe and its prevalence continues to grow [2, 3]. Even in the ablation era, electrical cardioversion (EC) remains a quick and effective method to convert AF to sinus rhythm [4]. Because most patients exceed 48 h or longer AF initiation, in most cases, EC needs to be scheduled after at least 3-week anticoagulation. In this period, pre-treatment with antiarrhythmic drugs can improve efficacy and facilitate EC [5].

Until nowadays there have been no strong predictors that allow physicians to identify the AF patient in whom to attempt EC with an acceptable probability of subsequent sinus rhythm maintenance. On this topic, a lot of various data among ECG measurements, echocardiographic parameters and biomarkers have been proposed, but no method has demonstrated good predictability so far [6, 7].

Some authors have suggested that atrial phasic function assessment can stratify the risk of AF post-EC [8]. This is further substantiated by the fact that (1) we know the potential relationship existing between risk of AF and a predisposing condition such as LV diastolic dysfunction [9] and (2) that the atrial conduit contribution to LV filling has been shown to be in direct relation with the degree of the underlying LV diastolic impairment in heart failure patients [10].

In this study, we demonstrate the existence of a strong association between the conduit contribution to LV filling volume, as computed with 3D echocardiography, and the arrhythmia recurrence in a group of patients with persistent AF and recent EC.

## Methods

### Patients’ population

After appropriate informed consent in agreement with institutional human review studies committee guidelines and local IRB approval, we enrolled prospectively 96 consecutive patients who underwent EC for persistent non-valvular AF. Patients younger than 18 years, with mitral prosthesis or severe mitral regurgitation and patients who did not respond to EC were, per protocol, excluded. To minimize possible bias related to various cycle duration differences during AF, patients were imaged few hours after successful EC, in regular sinus rhythm. During subsequent clinical follow-up all patients underwent clinical survey and a 24-h Holter ECG recording at 1 month, with AF recurrence defined as symptomatic or asymptomatic episode of atrial arrhythmia (> 30 s, registered AF or flutter or atrial tachycardia on ECG or Holter examination) causing cardiology consultation. The characteristics of the patients’ population (subdivided according to AF recurrence within 1 month) are reported in Table 1, identified by comorbidities and treatment in addition to conditions comprised within the CHA2DS2VASc score. We also reported other potential important clinical situations as described by Deng et al. [11] as part of new scores proposed for AF. All cardiovascular chronic therapy and in particular antiarrhythmic drugs were started, for all patients, after an appropriate anticoagulation period one week before scheduled EC.

**Table 1:** Patients’ clinical characteristics and pharmacological treatment

Characteristics	Total pts (no. = 96)	SR (no. = 62)	AF (no. = 34)
Age (years)	73 (67–77)	72.5 (66.8–76.3)	73 (66.8–78.3)
BMI (kg/m^2^)	28.4 ± 3.89	28.4 (25.9–30.8)	28.2 (25.5–31.3)
CHA2DS2VASc	3 (2–4)	3 (2–4)	3 (2–4)
Sex (M/F)	66/30	45/17	21/13
Underlined cardiopathy (no. of pts)			
Hypertensive	55	37	18
Hypertrophic	1	1	0
Post-ischaemic	3	3	0
Dilatative	5	2	3
Mild-reduced EF	3	2	1
Valvular	4	3	1
No other than AF	25	17	8
AF history duration (months)—unknown in 56 pts	4.9 ± 2.9	5.0 ± 2.5	4.8 ± 2.1
Oral medical treatment (no. of pts)			
Amiodarone	56	37	19
Propafenone	13	10	3
Flecainide	12	7	5
Sotalol	4	4	0
Digoxin	22	14	8
Beta-blockers			
Bisoprolol	47	31	16
Metoprolol	4	4	0
Carvedilol	4	2	2
Nebivolol	7	3	4
Atenolol	4	4	0
Ca^++^ channel blockers			
Verapamil	2	2	0
Ace inhibitors	31	22	9
MRA	20	13	7
VKA	74	52	22
NOA	22	10	12
Comorbidities (no. of pts)			
COPD	6	1	5
OSAS	4	3	1
CKD (GFR < 50 ml/min)	14	9	5
Metabolic disease	61	39	22
Current smoking	6	3	3
Bundle branch block	9	7	2
Hypertension	80	52	28
Diabetes	19	12	7

*AF* atrial fibrillation, *F* female, *CKD* chronic kidney disease, *COPD* chronic obstructive pulmonary disease, *EF* ejection fraction, *M* male, *MRA* mineralocorticoid receptor antagonist, *NOA* new oral anticoagulants, *OSAS* obstructive sleep apnea syndrome, *SR* sinus rhythm, *VKA* Vitamin K antagonist

### Echocardiographic examination

Transthoracic echocardiography data were acquired after successful EC (3 h ± 15 min) using a Vivid E9 (GE Medical Systems, Horten, Norway) system with both a 2D probe (average frame rate 65 frame/s) and a 4V probe for 3D acquisition (average 30 frame/s). We used apical four-chamber and two-chamber gray-scale and color views, with pulsed and continuous mitral flow velocity tracings. An apical four-chamber tissue Doppler acquisition was also obtained, together with a triplane apical LV volumetric view using the 4V transducer.

At the end of the standard examination, a multi-beat (at least five consecutive cardiac cycles) pyramidal 3D echocardiographic full-volume dataset was acquired from the apex in each patient. The volume data was displayed in real-time, two orthogonal apical views and three cross-sectional slices, with optional volume rendering techniques for visualization of valves and structures. Full-volume acquisition was obtained during held respiration, with dataset sector (frame rate ranging between 20 and 40 frames/s) dimensions and depth set to include both the ventricle and the atrium, assuring volume sampling rates between 15 and 25/s [10].

Subsequently, using a commercially available software package (EchoPAC PC version BT112, GE Healthcare), each view was aligned to the standard apical views, using the positions of the mitral annulus plane and of the apex as markers. Initialization was done at end-diastole (ED), using two clicks in each view (apex and mitral annulus plane), with contouring and 3D surface detection automatically drawn. Once completed for ED, the same procedure was used for ES, with LV endocardial border tracked throughout the entire cardiac cycle. Surface 3D detection was automatically triggered, and if necessary, the detected 3D surface was edited manually by adding landmark points. After both ED and ES images were finalized, full 4D surface detection was performed. The same procedure was followed for LA chamber using the mitral annular plane and the roof of the cavity as reference markers [10, 12, 13]. Finally, LV and LA volumes, along with their respective time–volume curves, were derived from the triangulated surfaces by summation of all triangular patches using the divergence theorem [10].

**Fig. 1:**
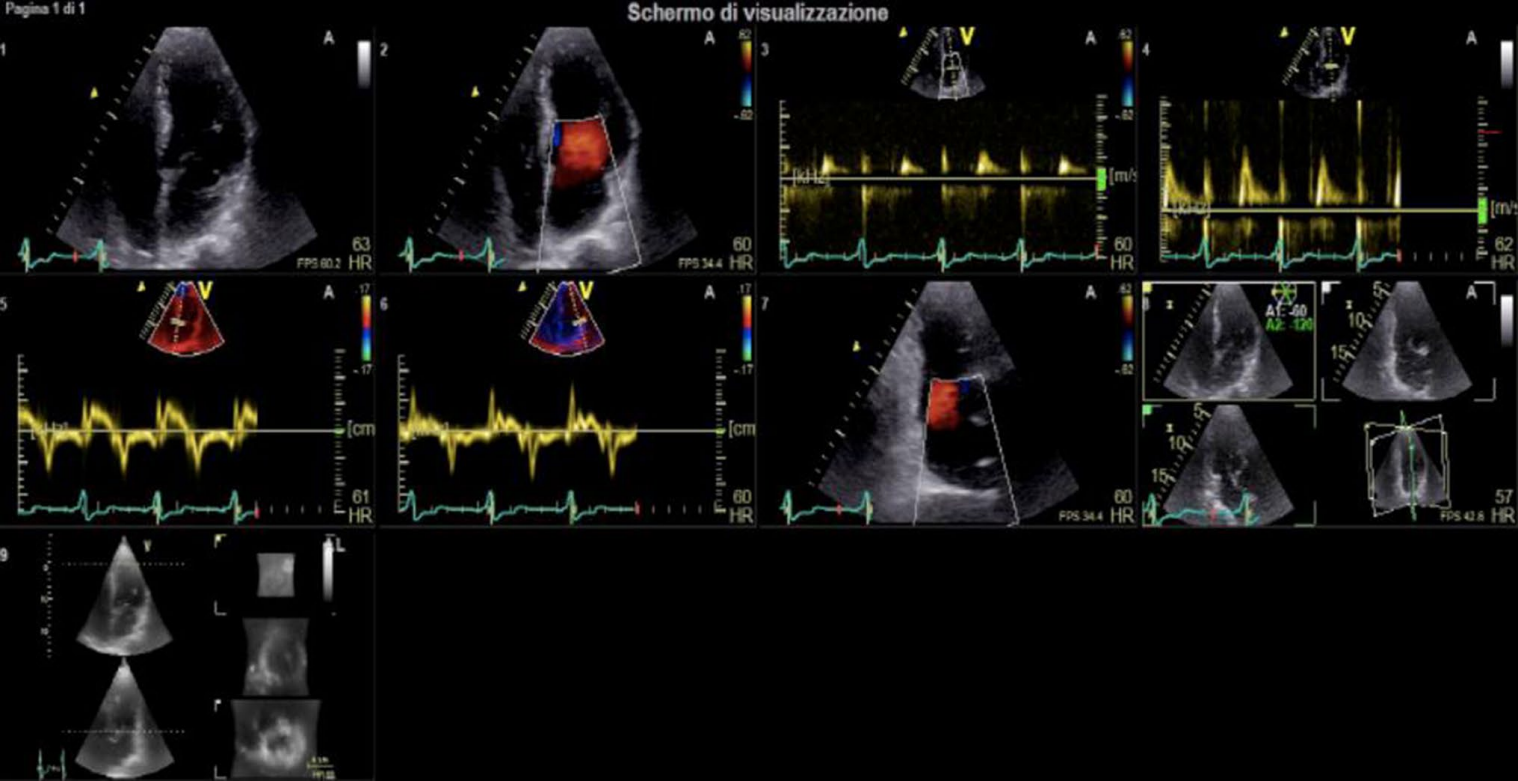
Dataset of echocardiographic acquisitions. Panel of different echocardiographic acquisitions with 2D and 3D probe

LA longitudinal strain was assessed using a 2D speckle-tracking technique from standard gray-scale loops. Regional deformation of six LA segments located along the perimeter of the LA cavity was assessed in the apical four-chamber view starting from the QRS complex, with an average of six equally spaced wall segments, generally peaking in late systole, as LA peak strain. Longitudinal LV strain was also assessed from the same images which were used to evaluate LA strain and it was averaged over six segments along the ventricular septum, apex, and lateral wall [14]. An example of the echo dataset used in each patient is presented in Fig. 1.

We, then, performed an off-line analysis using a commercially available spreadsheet program to transfer data and synchronize the LA and LV volume curves as a function of time. Early after EC, patients presented regular sinus rhythm at ECG surface but with echo characteristics of atrial mechanical stunning, as could be assumed from the null or very little lateral a’ peak wave at mitral tissue Doppler imaging [4 (3–5) cm/s)]. At this stage, we assumed that atrial phasic functions could be computed as

reservoir: maximum LAV − minimum LAV (LAV_max_− LAV_min_),

conduit: LV stroke volume – reservoir,

considering that pump should be negligible because of atrial stunning immediately post-EC. Phasic functions were then expressed as a percent of LV stroke volume. We also evaluated total LA emptying fraction (EF), computed as (LAV_max_ − LAV_min_)/ LAV_max_, as already described with cardiac magnetic imaging [15].

To better define ventricular diastolic properties, we used the method described by Klotz et al. [16] to assess noninvasively LV single-beat end-diastolic elastance (EDPVR) by fitting the exponential curve EDP = α x EDV^β^, where EDP is the LV end-diastolic pressure and EDV is the end-diastolic volume, with *α* and *β* as curve-fit parameters. In our population, EDV was obtained by selecting the maximum value from the 3D—full-volume LV data curve, synchronous with QRS at surface ECG, while EDP was derived using the equation EDP = 11.96 + 0.596 × *E*/*é*, as proposed by Ommen et al. [17]. As already suggested by Shwarzl et al. [18], to compare the whole position of the LV EDPVR, we used the calculated EDV at 30 mmHg EDP (EDV_30_), as a measure of LV capacitance.

Finally, we computed LA stress, as derived from LAV_min,_ obtained from the time–volume curve synchronous with QRS at surface ECG, and pressure from EDP, shaping the LA cavity as with a spherical surface (thickness of the wall assumed to be 2 mm) [19] according to the following formula: (stress = 1.35 × EDP × LAVmin/4 × wall thickness × (1 + (wall thickness/LAVmin))) [20].

### Statistical analysis

Data are expressed as mean ± 1SD or median [25th–75th confidence intervals] if data were not normally distributed. Differences between means were assessed by unpaired t tests. A Mann–Whitney rank sum test was used if data were not normally distributed. A multiple logistic regression model was used to test the relationship between LA phasic conduit function, on top of other echocardiographic and biometric measurements, and early AF recurrences. Least square regression analysis was used as necessary. To find a diagnostic cut-off value of conduit for identification of short-term AF recurrence after EC, nonparametric receiver-operating characteristics (ROC) curve analyses were performed and the area under the curve showing the discriminatory ability of the cut-off variable reported. Sensitivity and specificity values of the best cut-off variable were also calculated. A *p* value < 0.05 was considered significant. Statistical analyses were performed using SigmaPlot version 12.5 for Windows statistical software (Jandel; San Rafael, CA, USA).

## Results

Results for the entire patients’ population and after they have been subdivided according to AF recurrence within 1 month are reported in Table 2. At 1-month follow-up, 62 patients (65%) were in sinus rhythm and 34 (35%) in AF. AF patients exhibited higher LA wall stress (*p* = 0.002) and *E*/*é* (*p* = 0.012), for non-dissimilar LA volumes (maximum: *p* = 0.383 and minimum: *p* = 0.07), but a stiffer LV cavity, as documented by a higher *β* value (*p* = 0.012) for a comparable capacitance (*p* = 0.461, Table 2). Conduit contributed more to LV stroke volume (*p* < 0.001, Table 2) in the AF subpopulation. On the contrary, LA EF was lower in this subgroup (*p* < 0.001), as obviously did reservoir function (Table 2). E wave peaked higher in AF group (*p* = 0.0136), thus modulating a larger *E*/*A* ratio, whose difference between the two sub-populations, however, did not reach statistical significance (*p* = 0.063).

**Table 2:** Post-cardioversion instrumental data for the entire patients’ population and after it has been divided according to 1-month recurrence of AF

Parameters	Total pts	SR (no. = 62)	AF (no. = 34)	*p*
HR (beat/min)	84.7 ± 19.0	80.0 [71.0–99.3]	82.0 [70.0–95.5]	0.583
LA stress (g/cm^2^)	129.9 ± 22.4	124.8 ± 21.6	139.2 ± 21.2	0.002
Systolic blood pressure (mmHg)	132.9 ± 15.1	133.8 ± 15.3	131.3 ± 14.9	0.455
Diastolic blood pressure (mmHg)	81.6 ± 10.1	81.3 ± 10.1	82.1 ± 10.2	0.723
Pulse pressure (mmHg)	51.4 ± 11.4	52.6 ± 11.9	49.2 ± 10.4	0.191
LV EDV (ml)	98.8 ± 26.5	96.6 ± 26.3	102.3 ± 26.7	0.344
LV EF (%)	51.7 ± 6.8	52.3 ± 6.3	50.7 ± 7.5	0.275
LAV min (ml)	52.2 ± 16.7	49.9 ± 16.6	56.4 ± 16.3	0.067
LAV max (ml)	65 ± 18.1	65.5 ± 18.8	64.1 ± 17.0	0.383
*E*/*é*	9.3 ± 3.8	7.43 (6.26–10.32)	10.27 (8.27–12.0)	0.012
*e*′ (cm/s)	10.1 ± 2.6	10.19 ± 2.60	9.85 ± 2.71	0.547
*a*′ (cm/s)	4.0 (3.0–5.0)	4.0 (3.0–6.0)	4.0 (3.0–5.0)	0.084
LA strain (%)	19.2 ± 6.6	19.3 (15.0–22.7)	17.6 (13.9–21.5)	0.408
LV strain (%)	− 14.4 ± 4.1	− 14.30 ± 3.91	− 14.60 ± 4.36	0.408
Conduit (%)	76.4 (68.2–81.8)	72.6 (62.0–76.4)	84.6 (79.8–89.5)	< 0.001
Reservoir (%)	23.6 (18.2–31.8)	27.4 (23.6–38.0)	15.4 (10.5–20.2)	< 0.001
LA EF (%)	20.2 ± 9.7	22.6 (18.7–24.5)	11.8 (9.5–16.0)	< 0.001
E (cm/s)	88.6 ± 25.5	83.9 ± 23.8	97.2 ± 25.7	0.014
A (cm/s)	31 (25.0–43.0)	32.0 (25.3–41.0)	30.5 (25.0–43.8)	0.455
*E*/*A*	2.8 ± 1.2	2.4 [1.8–3.2]	3.1 (1.9–4.3)	0.063
*β* (–)	6.08 (6.02–6.22)	6.05 (6.01–6.16)	6.15 (6.07–6.25)	0.012
*α* (x 10^12^)	8.9 (6.0–6.2)	9.7 (4.1–5.3)	4.4 (2.3–2.9)	0.058
LV (ml) capacitance_30_	108.2 ± 29.5	106.5 ± 29.5	111.2 ± 29.8	0.461

*AF* atrial fibrillation, *BMI* body mass index, *EDV* end–diastolic volume, *EF* ejection fraction, *HR* heart rate, *LA* left atrial, *LA EF* left atrial emptying fraction, *LAV* left atrial volume, *LV* left ventricle, *Pts* patients, *SR* sinus rhythm

Parameters significantly different at baseline between the 1 month AF versus sinus rhythm groups were used in the multiple logistic regression to test the existence of an independent predictor of AF recurrence short-term after EC. To minimize the problem of collinearity peak E wave value was not included, being already represented within the *E*/*é* ratio.

**Fig. 2:**
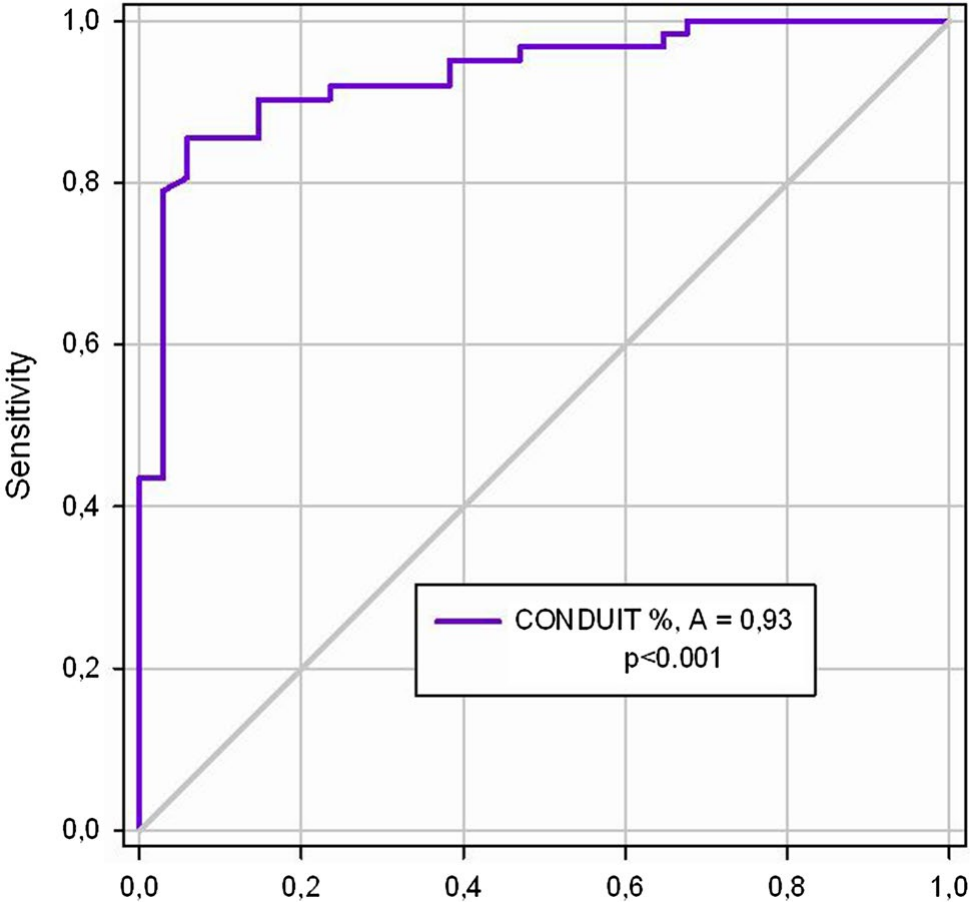
ROC curve for the prediction of early arrhythmia recurrence. A conduit value of 79% of ventricular stroke volume can identify those patients that reverted to AF early after EC (ROC area 0.93, *p* < 0.001) with 90% sensitivity and 85% specificity

Results of the multiple logistic regression are shown in Table 3. Conduit was the only significant predictor of short-term AF recurrence post-EC. Results were unmodified when patients’ age, BMI, use of amiodarone or class IC drugs were included in the model (Supplementary Table 1). Using ROC curve analysis (Fig. 2), we identified a conduit value of 79% of LV stroke volume as capable of identifying those patients that will revert to AF early after EC (ROC area 0.93, *p* < 0.001) with optimal sensitivity (90%) and specificity (85%).

**Table 3:** Multiple logistic regression

Independent variable	Coefficient	Standard error	*p* value
Constant	− 14.49	11.19	0.196
LA wall stress	− 0.004	0.032	0.891
*E*/*é*	0.264	0.178	0.138
Conduit	0.210	0.085	0.013
LA EF	− 0.189	0.108	0.081
*β*	− 0.168	1.108	0.880

*LA* left atrial, *LA EF* left atrial emptying fraction

Interobserver reproducibility for conduit has already been reported [10].

## Discussion

In the present study, we observed a strong relationship between the conduit atrial phasic function and arrhythmia recurrence early after EC in patients with persistent AF. This association suggests that information obtainable from the LAC computation does provide additional insight into the mechanical characteristics of the cavity and potentially stroke risk stratifies patients post-EC [21].

It is well known that LA modulates LV filling and function in three distinct phases: as a reservoir for pulmonary venous return during ventricular systole, as a conduit from pulmonary veins directly into the LV chamber during early ventricular diastole, and as a pump that seals ventricular filling at the end of the cardiac cycle [22]. In patients with AF the booster pump function is absent, while the other two phases are modified in a reciprocal way. Thus, in the immediate post-EC period, the amount of conduit favoring ventricular filling is reflected by the reciprocal change in reservoir contributing to stroke volume. The comment is not pleonastic: the emphasis on the conduit phase in relation to AF recurrence early after EC does delineate the concomitant, underlying LV diastolic dysfunction as the fundamental condition promoting AF recurrence in our patients’ population. The focus on the reservoir phase would point, instead, toward the atrial stiffening milieu as the principal cause for the recurrence of the arrhythmia [23–25].

The absence of any significant relationship, in the multivariate analysis, between the LV β stiffness coefficient and the recurrence of the arrhythmia points, but not exclusively, in favor of the second interpretation. Previous nuclear magnetic imaging data are indeed available that associate the reservoir function to the LA enhancement in AF patients, suggesting that those with AF have more structural changes as compared with healthy volunteers [15]. Furthermore, AF is associated with different alterations in LA characteristics that permit the progression and persistence of the arrhythmia itself. We assist to an adaptation of the atrial refractory potentials and to a modification of the contractile properties with a loss of the atrial contraction (pump function) that itself promotes chamber dilatation [26]. This chamber dilation, according to the law of La Place, implies a linear increase in wall tension so as LA diameter increases atrial wall stress increases too [9].

It must also be considered that the duration of the AF episode may affect LA conduit function post-EC through its influence on chamber remodeling pre-EC. In this regard, we could demonstrate a significant direct linear relationship between conduit (expressed in ml) and LA minimal and maximal cavity volume (*r* = 0.35 *p* < 0.001 and *r* = 0.23 *p* < 0.03, respectively). No relation, instead, could be demonstrated between conduit and AF duration pre-EC (*r* = 0.13 *p* = 0.53). However, this can be in part due to the limited number of patients for whom the arrhythmia duration was known (27/96). For this reason, besides the unavailability of echo data post-EC, we cannot confirm or exclude a potential modification of conduit in the first few weeks after EC due to the influence of a positive chamber remodeling that may take place in that same period.

### Atrial phasic function, strain and volume

Most studies on LA diastolic function have used the echocardiographic speckle-tracking technique to assess strain and indirectly assess cavity compliance [27], while other authors have investigated the atrial diastolic function by means of nuclear magnetic imaging, relying entirely on LA time–volume curve changes [28]. There are caveats for both approaches. First, if one uses speckle-tracking analysis he must accept the well-known limitation that there are regional differences in the LA segmental function during atrial contraction and relaxation. The posterior wall, in fact, exhibits the lowest strain because of the attachment of the pulmonary veins, while the inferior wall generates the highest deformation, because of its greater thickness [29]. Second, a deformation gradient is detectable from all views during atrial contraction and relaxation, with the generation of a higher strain at the atrioventricular junction and a lower strain in the atrial roof because this is fixed to the mediastinum [29]. Therefore, any segmental model ignoring posterior wall could overestimate global strain. Similarly, the four-chamber view of LA incorporates the interatrial septum and the area of the pulmonary veins, in which atrial strain is low. Thus, model variability is one of the possible conflicts and a major reason of such different cut-off values as reported in clinical diseases [30]. As far as LA volume curve changes are concerned, it has to be remembered that a correct assessment of the atrial phasic function from such a single time–volume curve is questionable [31].

Therefore, we think that the estimation of the cavity conduit function, expressed in term of percentage volumes that can be comprehensively obtained by simultaneous atrioventricular 3D acquisition of full-volume data sets, could better describe alteration of the atrial-ventricular coupling which characterizes the condition predisposing to AF recurrence and to document the presence and the degree of any coexistent LV diastolic dysfunction, when present.

We have already demonstrated that conduit contribution to LV filling increases with worsening of diastolic dysfunction [10] and that such parameter could be proposed for evaluation of diastole [32]. It is important to note that other authors, using cardiac computed tomography, have similarly demonstrated that diastolic dysfunction is characterized by increasing of conduit volume [33].

In our study, conduit, and obviously its reciprocal reservoir, represents a strong independent predictor of AF recurrence in a situation in which the confounding effect of the atrial booster pump function is minimized, like in the phase immediately following EC.

## Limitations

Our study presents some limitations mainly represented by the short length of follow-up after EC (1 month). While recognizing the shortness of such interval we must underline the fact that most of the recurrences take place shortly after EC [34]. In any case, it would be interesting to extend the follow-up for a longer period of time to gather further confirmation of the original hypothesis, beyond the protocol so far completed.

We have chosen the time immediately after EC to acquire all the echocardiographic data, thus obviously limiting the clinical implications of our findings on the everyday clinical practice, when patients are imaged generally before EC. This was done to obtain data in regular sinus rhythm, minimizing the problem that could derive from the irregular R–R intervals that characterize the AF condition preceding EC in these patients. This consideration, however, does not exclude, for the future, the idea of imaging patients before EC. As the focus of the pathophysiological reasoning relies on the atrio-ventricular coupling process, we are convinced that the variations in the R-R intervals, which characterize AF, cannot substantially obscure the information on which the prediction is based.

This is a single-center, small-sized cohort study that needs to be reproduced and verified in a larger population possibly studied across different centers.

## Conclusions

Conduit phasic function is a good descriptor of diastolic derangement relating both LA and LV volumes in particular during AF, when the pump is lost. The increment in conduit, expressed relative to LV stroke volume, and the consequent reduction in reservoir function due to the reciprocation of the two phases in our model reflects a context of increased atrioventricular stiffness, a condition prone to be a strong predictor of AF early recurrence after EC. Whether this analysis can also provide potential better stroke risk stratification in patients’ post-EC will be the object of a future study.

## Electronic supplementary material

Below is the link to the electronic supplementary material.

Supplementary material 1 (DOCX 14 KB)
