# Supplementary material for: Association between left atrial phasic conduit function and early atrial fibrillation recurrence in patients undergoing electrical cardioversion
Source: Clin Res Cardiol. 2017 Nov 27;107(4):329–37. doi: 10.1007/s00392-017-1188-9 (PMC5869942; doi:10.1007/s00392-017-1188-9)
Supplement: Supplementary file 1 — Supplementary material 1 (DOCX 14 KB) [file 392_2017_1188_MOESM1_ESM.docx]

Table 1 (supplement). Multiple logistic regression (supplement)

*Abbreviations* : *BMI*=body mass index, *LA*=left atrial, *LA EF*=left atrial emptying fraction

| *Indepedent variable* | *Coefficient* | *Standard Error* | *p-value* |
| --- | --- | --- | --- |
| Constant | -26.69 | 14.03 | 0.06 |
| Age | 0.094 | 0.06 | 0.131 |
| BMI | 0.0005 | 0.104 | 0.960 |
| Amiodarone (yes/no) | -0.539 | 1.222 | 0.659 |
| IC drugs (yes/no) | 1.123 | 1.482 | 0.448 |
| LA wall stress | -0.0001 | 0.035 | 0.998 |
| E/é | 0.230 | 0.202 | 0.253 |
| Conduit | 0.252 | 0.094 | 0.008 |
| LA EF | -0.184 | 0.120 | 0.125 |
| β | 0.104 | 1.131 | 0.927 |

.
